# Evaluation of the clinical efficacy of 3D Printing technology assisted surgery combined with early postoperative comprehensive rehabilitation in the treatment of Senile Intertrochanteric Fractures

**DOI:** 10.12669/pjms.37.3.3988

**Published:** 2021

**Authors:** Ye Zhao, Yong-hui Liu, Shu-ge Mao, Xin-xin Zhang

**Affiliations:** 1 Ye Zhao, Department of Orthopedics, Henan University of Chinese Medicine, Zhengzhou, Henan 450000, P.R.China; 2 Yong-hui Liu, Department of Orthopedics, Henan University of Chinese Medicine, Zhengzhou, Henan 450000, P.R.China; 3 Shu-ge Mao, Department of Orthopedics, Luoyang Orthopedic-Traumatological Hospital of Henan Province (Henan Provincial Orthopedic Hospital), Luoyang, Henan 471000, P.R.China; 4 Xin-xin Zhang, Department of Orthopedics, Luoyang Orthopedic-Traumatological Hospital of Henan Province (Henan Provincial Orthopedic Hospital), Luoyang, Henan 471000, P.R.China

**Keywords:** 3D printing, Early comprehensive rehabilitation, Elderly, Intertrochanteric fracture, Treatment

## Abstract

**Objectives:**

To observe the clinical value of 3D printing technology assisted surgery combined with early postoperative comprehensive rehabilitation in elderly patients with intertrochanteric fractures.

**Methods:**

Sixty elderly patients with intertrochanteric fractures of the femur who were treated in our hospital from January 2018 to January 2020 were selected and randomly divided into two groups. In the experimental group, 3D printing technology assisted surgery combined with early postoperative comprehensive rehabilitation was used for treatment. While in the control group, traditional open reduction and dynamic hip screw internal fixation combined with postoperative conventional treatment was utilized. The duration of surgery, intraoperative blood loss, postoperative hospital stay, weight bearing time, fracture healing time and other surgical indicators were recorded respectively, and hip joint function recovery was evaluated prior to and 2 weeks after surgery. All patients were followed up for six months to observe the occurrence of complications within half a year, including deep vein thrombosis, incision infection, avascular necrosis of femoral head, hip joint stiffness, delayed fracture healing, etc. Subsequently, the differences in postoperative complications between the two groups were compared and analyzed.

**Results:**

The operation time, blood loss, postoperative hospital stay, weight bearing time and fracture healing time of the experimental group were better than those of the control group, and the difference was statistically significant (p<0.05). After treatment, the hip joint function of the experimental group was significantly improved compared with the control group, with a statistically significant difference(p=0.03). The incidence of operative complications in the experimental group was 10% (3/30) within six months postoperatively, significantly lower than the 33% (10/30) in the control group, with statistical significance (p=0.03).

**Conclusion:**

3D printing with early rehab proved to be effective treatment in our study. Such a combined treatment has the advantages of precise operative reduction, fast postoperative recovery, and certain safety and effectiveness.

## INTRODUCTION

Intertrochanteric fracture, as a clinically common type of fracture that occurs more frequently in women,^1^ will affect about 18% of females and 6% of males according to analysis.^2^ Patients suffering from intertrochanteric fractures are in a dangerous condition and need to be actively treated with surgery-based comprehensive treatment, otherwise, their quality of life will be severely affected or even their lives will be threatened.^3^ The accurate surgical reduction is the key to the success of treatment, but certain benefits will be brought about by postoperative comprehensive treatment to improve patients’ quality of life.^4^

3D printing technology is an emerging surgical method applied in clinical operations in recent years. Stereoscopic models can be printed out by preoperative CT, MRI and other technology imaging as well as later computer 3D imaging, so that the type and location of fracture can be more accurately evaluated, and an intuitive physical model can be provided for formulating a more detailed plan for surgery.^5^ 3D printing technology is conducive to precise surgical reduction by virtue of the above-mentioned performance and advantages. It has been reported that the application of 3D models in orthopedic surgery can benefit patients by reducing surgery time and blood loss.^6^ Establishing a 3D model of the spine for patients with spinal deformity before surgery can help reduce the operation time, reduce the radiation exposure to the patient during the operation, and improve the overall surgical outcome.^7^ The concept of early postoperative comprehensive rehabilitation exerts its unique role in reducing the psychological and physiological stress trauma of patients, and significantly promoting the recovery of motor function and rehabilitation quality of patients.^8^

This study aims to explore the application value of 3D printing technology combined with early comprehensive rehabilitation treatment for elderly patients with femoral intertrochanteric fracture surgery. The specific research details are now reported as follows.

## METHODS

A total of sixty elderly patients with intertrochanteric fractures of the femur who were treated in our hospital from January 2018 to January 2020 were selected.

### Ethical approval:

The study was approved by the Institutional Ethics Committee of Luoyang Orthopedic-Traumatological Hospital of Henan Province(Henan Provincial Orthopedic Hospital) (No.2018-09), and written informed consent was obtained from all participants.

### Inclusion criteria:

(1) Patients aged > 60 years; (2) Patients diagnosed with an intertrochanteric fracture after preoperative imaging examination;^9^ (3) Patients without local trauma or local bone-related diseases in the past; (4) Be voluntarily recruited to the research and sign the consent form; (5) No obvious mental disorder, able to cooperate in completing the research work.

### Exclusion criteria:

(1) Combined with other fractures; (2) Patients with severe mental disorders who cannot cooperate to complete the research; (3) Patients with other severe underlying diseases that cannot be corrected and who are unable to tolerate surgery. sixty patients were randomly divided into two groups with 30 patients in each group. In the experimental group, 3D printing technology assisted surgery combined with early postoperative comprehensive rehabilitation was used for treatment. While in the control group, traditional open reduction and dynamic hip screw internal fixation combined with postoperative conventional treatment was used. 16 females and 14 males were enrolled in the experimental group, aged 60 - 75 years, with an average age of 67.69±4.27 years old. 18 females and 12 males were enrolled in the control group, aged 63 - 73 years, with an average age of 68.58±3.41 years old. There was no significant difference in general information between the two groups (p>0.05), which were comparable (Table-I).

**Table-I:** Comparative analysis of general information of the two groups (χ̄±S) n=30.

Indicators	Experimental group	Control group	t/χ^2^	p
Male (%)	14(47%)	12(40%)	0.31	0.66
Female (%)	16(53%)	18(60%)	0.27	0.60
Mean Age	67.69±4.27	68.58±3.41	0.89	0.38

p>0.05.

### Surgical Method:

Traditional open reduction and dynamic hip screw internal fixation was used in the control group: patients were given epidural anesthesia or general anesthesia. After the anesthesia was successful, the patient was placed in a horizontal position with a thin pillow under the buttock of the affected side, and routine disinfection and draping were carried out. An incision about 15cm long was made at the top of the femoral greater trochanter on the affected side. The skin, subcutaneous tissue and bluntly separated muscle layer were incised layer by layer to fully expose the greater trochanter and lateral aspect of proximal femur The fracture was then reduced under direct vision, and traction was maintained after satisfactory reduction. Under the C-arm fluoroscopy, the guide pin was inserted 2.5 cm below the greater trochanter on the outside of the femur. After the guide pin was confirmed to be located in the middle of the femoral neck and head, the length of the guide pin was measured, the holes were reamed along the guide pin, and the steel plates and screws were placed and fixed under pressure. Finally, the wound was cleaned with sterile saline and sutured layer by layer.

### 3D printing technology assisted surgical reduction:

3D printing technology assisted surgical reduction was applied in the experimental group for reduction. Patients were scanned by CT or MRI at the fracture site before surgery, and the scanning data were input into imaging processing software for processing, and an equal-proportion intertrochanteric fracture model was made by 3D printing technology. The specific scheme of fracture reduction was designed by surgeons based on the model, and the surgical simulation exercise was carried out by using 3D printing entities. After the simulation matured, the surgery of the experimental group was carried out according to the surgical scheme of the control group.

### Early comprehensive rehabilitation:

Patients in the control group were given routine treatment postoperatively, including sedation, pain relief, nutrition treatment and rehabilitation exercise. Patients in the experimental group were given early postoperative comprehensive rehabilitation treatment, and an individualized comprehensive rehabilitation scheme was formulated for different patients. The following contents are covered in the scheme: (1) Early start of isometric contraction training of lower limb muscles. (2) Early implementation of active and passive activities such as hip joint, knee joint and ankle joint of the affected side; (3) Active and passive stretching of soft tissues such as ligaments, tendons and muscles around the affected side of the hip joint under the condition that the internal fixation is stable and not displaced, with the force of the stretching controlled according to the patient’s tolerance. (4) Strengthening of physical rehabilitation therapy, such as low and medium frequency electrical stimulation, lower extremity air pressure therapy to promote blood circulation.

### Observation indicator Surgical indicator:

(1) Differences in the duration of surgery, intraoperative blood loss, postoperative hospital stay, weight bearing time, fracture healing time and other surgical indicators between the two groups were recorded separately. (2) The hip joint function was evaluated before and 2 weeks after surgery and the recovery of hip joint function before and after surgery was analyzed. The Harris Hip Joint Function Scale was adopted for the hip function score.^10^ On a 100-point scale, a score of 90 points or more is considered as an excellent outcome; 80-89 points, a good outcome; 70-79 points, a fair outcome; and 70 points or less, a poor outcome.

### Complication indicator:

The complications of the two groups within six months were recorded respectively, including deep vein thrombosis, incision infection, avascular necrosis of the femoral head, hip joint stiffness, delayed fracture healing, etc. Subsequently, the differences in postoperative complications between the two groups were compared and analyzed. The follow-up work of all patients was completed by the same group of surgeons.

### Statistical analysis:

All the data were statistically analyzed by SPSS 20.0 software, and the measurement data were expressed as (χ̄ ± s). The data between the experimental group and the control group were analyzed by an independent sample t-test, while the data of the experimental group and the control group before and after treatment were analyzed by paired t-test, and the comparison of the rate was conducted by the chi-square test. P<0.05 indicates a statistically significant difference.

## RESULTS

As indicated by the comparison of surgery conditions between the two groups, the experimental group was superior to the control group in terms of duration of surgery, blood loss, postoperative hospital stay, weight bearing time and fracture healing time, with statistically significant differences (duration of surgery, blood loss, weight bearing time and fracture healing time, p=0.00; postoperative hospital stay, p=0.03) (Table-II).

**Table-II:** Comparative analysis of the two groups of surgery (χ̄±S) n=30

Group	Duration of surgery (min)	Blood loss (ml)	Postoperative hospital stay (d)	Weight bearing time (d)	Fracture healing time (weeks)
Experimental group	74.34±15.26	165.31±24.34	16.86±4.73	53.23±3.27	11.35±2.16
Control group	124.76±13.12	277.35±15.82	20.17±6.53	67.49±7.63	15.58±1.44
t	13.72	21.14	2.25	9.41	8.92
p	0.00	0.00	0.03	0.00	0.00

p<0.05.

The comparison of hip joint function scores (Harris scores) between the two groups before and after treatment is shown in Table-III. There were no significant differences in Harris scores between the experimental group and the control group before treatment. After 2 weeks of treatment, Harris scores of the two groups significantly improved compared with that before treatment (p=0.00), and that of the experimental group was significantly higher than that of the control group, with a statistically significant difference (p=0.03).

**Table-III:** Comparative analysis of hip joint function scores between the two groups before and after surgery (χ̄±S) n=30

	Before treatment	After treatment	t	p
Experimental group	63.32±7.83	83.44±12.35	7.54	0.00
Control group	65.18±8.43	76.79±10.68	4.67	0.00
t	0.89	2.23		
p	0.38	0.03		

p<0.05.

**Table-IV:** Comparative analysis of the incidence of complications between the two groups (χ̄±S) n=30

Indicators	Deep venous thrombosis	Incision infection	Femoral head necrosis	Joint stiffness	Delayed fracture healing	Total
Experimental group	0	1	0	0	2	3(10%)
Control group	1	1	0	6	2	10(33%)
χ^2^						4.36
p						0.03

p<0.05.

As indicated by the comparison of postoperative complications between the two groups within half a year, there were one case of incision infection and two cases of delayed fracture healing in the experimental group, with a complication rate of 10% (3/30). While in the control group, the incidence of surgical complications was 33% (10/30). No serious complications occurred between the two groups. The incidence of complications in the experimental group was significantly lower than that in the control group (p=0.03).

## DISCUSSION

Clinically, the incidence of intertrochanteric fractures is relatively high, mostly in middle-aged and elderly people. Middle-aged and elderly people are at risk of osteoporosis and may suffer from intertrochanteric fractures due to accidental falls or bumps, some of which are not exposed to great external forces.^9^ With the increasingly serious aging of the population, the incidence rate is increasing year by year.^11^ The higher disability rate is caused by intertrochanteric fractures, so that the quality of life of patients is seriously affected and the survival rate is greatly reduced.^12^ Despite a relatively abundant intertrochanteric blood circulation and easier healing after treatment, serious pathological consequences may be caused to patients due to unsatisfactory reduction and healing.^13^

It is believed by research conducted by Lv et al that traditional technology is not particularly reliable. Accurate sample production and reliable quantitative analysis of trabeculae between individuals can be realized with the aid of computer tomography and 3D equipment, thus providing a more objective method for better anatomical reduction.^14^ Preoperative evaluation and pre-surgical operation using the 3D printing model^15^ is conducive to confirming the sequence of reduction and fixation, determining the quality of reduction, shortening the duration of surgery and minimizing the difficulty of surgery.^16^ It is also believed by Chen et al. that the pre-experiment of the 3D printing template is conducive to the accurate placement of the intramedullary needle during surgery.^17^ It can be concluded from our research that the experimental group is superior to the control group in terms of duration of surgery, blood loss and postoperative hospital stay (duration of surgery, blood loss, p=0.00; postoperative hospital stay, p=0.03) with the aid of 3D printing technology assisted surgery. The hip function score of the experimental group was significantly better than that of the control group (p=0.03), suggesting that the preoperative examination and preoperative imaging preparation were slightly complicated due to the integration of 3D printing technology, but the quality of surgery was significantly improved compared with traditional surgery.

Fast-track surgery (FTS) was first proposed by a Danish anesthesiologist Dr. Henrik Kehlet in 2001,^18^ with the main point of its implementation being the application of optimized measures proven by evidence-based medicine in the perioperative period to reduce surgical stress response and postoperative complications.^19^ A variety of perioperative nursing measures and other interventions are also included in FTS, which can shorten the length of stay, reduce the cost of hospitalization, and promote the rapid recovery of patients. Quite a few pieces of pieces of evidence have shown that the length of hospital stay can be significantly shortened and the satisfaction of patients with postoperative complications can be improved via the widespread application of FTS.^20^ FTS concept, compared with traditional perioperative measures, has its application in surgery to significantly enhance the recovery ability of various organs and functions of patients.^21^ It is believed by Mingli et al., that the average Harris hip score of preoperative and postoperative patients was 85 and 47 respectively after the application of the FTS concept and traditional surgical postoperative care, with an excellent rate of 82.7%.^22^ It was shown in our research that the patients who applied the individualized FTS concept in the early postoperative period recovered significantly faster than the control group, and the weight bearing time and fracture healing time were shorter than the control group, with a significant difference (p =0.00). At the same time, the incidence of postoperative complications was significantly lower than that of the control group (p=0.03), especially in deep vein thrombosis and joint stiffness.

### Limitations of the study:

The shortcomings of this research lie in that the long-term effects cannot be objectively evaluated owing to the small number of patients and the short follow-up time. In addition, for certain indicators such as fracture healing time, weight bearing time and hip function recovery, it has not been clearly distinguished and elaborated whether the above-mentioned changes are caused by the guiding significance of 3D printing in the surgery or by the implementation of early postoperative rehabilitation surgical measures, or whether an equally important role is played by both.

## CONCLUSIONS

To sum up, 3D printing with early rehab proved to be effective treatment in our study. Such a combined treatment has the advantages of precise surgical reduction, fast postoperative recovery, and certain safety and effectiveness.

### Authors’ Contributions:

**YHL & YZ:** Designed this study and prepared this manuscript, are responsible and accountable for the accuracy or integrity of the work.

**XXZ:** Collected and analyzed clinical data.

**SGM:** Significantly revised this manuscript.

## References

[1] Barrios-Moyano A, Contreras-Mendoza EG (2018). Frecuencia de complicaciones en pacientes mayores de 60 años con fractura de cadera [Frequency of complications in patients older than 60 years with hip fracture]. Acta Ortop Mex.

[2] Veronese N, Maggi S (2018). Epidemiology and social costs of hip fracture. Injury.

[3] Alexiou KI, Roushias A, Varitimidis SE, Malizos KN (2018). Quality of life and psychological consequences in elderly patients after a hip fracture:A review. Clin Interv Aging.

[4] Aftab A, Awan WA, Habibullah S, Lim JY (2020). Effects of fragility fracture integrated rehabilitation management on mobility, activity of daily living and cognitive functioning in elderly with hip fracture. Pak J Med Sci.

[5] Marongiu G, Prost R, Capone A (2019). A New Diagnostic Approach for Periprosthetic Acetabular Fractures Based on 3D Modeling:A Study Protocol. Diagnostics (Basel).

[6] Weidert S, Andress S, Suero E, Becker C, Hartel M, Behle M (2019). 3D printing in orthopedic and trauma surgery education and training:Possibilities and fields of application. Unfallchirurg.

[7] Zhang RF, Wang PY, Yang M, Dong XB, Liu X, Sang YG (2019). Retracted Article:Application of 3D printing technology in orthopedic medical implant - Spinal surgery as an example. Int J Bioprint.

[8] Su B, Newson R, Soljak H, Soljak M (2018). Associations between post-operative rehabilitation of hip fracture and outcomes:national database analysis (90 characters) [published correction appears in BMC Musculoskelet Disord.

[9] Horwitz DS, Tawari A, Suk M (2016). Nail Length in the Management of Intertrochanteric Fracture of the Femur. J Am Acad Orthop Surg.

[10] Kumar P, Sen R, Aggarwal S, Agarwal S, Rajnish RK (2019). Reliability of Modified Harris Hip Score as a tool for outcome evaluation of Total Hip Replacements in Indian population. J Clin Orthop Trauma.

[11] Karakus O, Ozdemir G, Karaca S, Cetin M, Saygi B (2018). The relationship between the type of unstable intertrochanteric femur fracture and mobility in the elderly. J Orthop Surg Res.

[12] Nasab SAM, Khorramdin E (2017). The assessment of mortality and quality of life after intertrochanteric fracture of femur in patients older than 60 at Emam Khomeini Hospital of Ahvaz. Pak J Med Sci.

[13] Yuan BJ, Abdel MP, Cross WW, Berry DJ (2017). Hip Arthroplasty After Surgical Treatment of Intertrochanteric Hip Fractures. J Arthroplasty.

[14] Lv H, Zhang L, Yang F, Li M, Yin P, Su X (2015). A novel 3D-printed device for localization and extraction of trabeculae from human femoral heads:a comparison with traditional visual extraction. Osteoporos Int.

[15] Manganaro MS, Morag Y, Weadock WJ, Yablon CM, Gaetke-Udager K, Stein EB (2017). Creating Three-dimensional Printed Models of Acetabular Fractures for Use as Educational Tools. Radiographics.

[16] Liu ZJ, Jia J, Zhang YG, Tian W, Jin X, Hu YC (2017). Internal Fixation of Complicated Acetabular Fractures Directed by Preoperative Surgery with 3D Printing Models. Orthop Surg.

[17] Chen X, Chen X, Zhang G, Lin H, Yu Z, Wu C (2017). Accurate fixation of plates and screws for the treatment of acetabular fractures using 3D-printed guiding templates:An experimental study. Injury.

[18] Otero JJ, Detriche O, Mommaerts MY (2017). Fast-track Orthognathic Surgery:An Evidence-based Review. Ann Maxillofac Surg.

[19] Pollmann CT, Røtterud JH, Gjertsen JE, Dahl FA, Lenvik O, Årøen A (2019). Fast track hip fracture care and mortality - an observational study of 2230 patients. BMC Musculoskelet Disord.

[20] Zanker J, Duque G (2017). Rapid Geriatric Assessment of Hip Fracture. Clin Geriatr Med.

[21] Grant MC, Yang D, Wu CL, Makary MA, Wick EC (2017). Impact of Enhanced Recovery After Surgery and Fast Track Surgery Pathways on Healthcare-associated Infections:Results From a Systematic Review and Meta-analysis [published correction appears in Ann Surg.

[22] Mingli F, Huiliang S, Guanglei C, Zheng L, Shibao L, Limin L (2017). A clinical study on arthroplasty for failed internal fixation of hip fractures and review of literature. Pak J Med Sci.

